# The Impact of Social Support on Subjective Cognition Across Adulthood

**DOI:** 10.1093/geroni/igaa057.1986

**Published:** 2020-12-16

**Authors:** Annalee Mueller, Jillian Minahan, Karen Siedlecki

**Affiliations:** 1 Fordham University, Bronx, New York, United States; 2 Fordham University, New York, New York, United States

## Abstract

Increased age is associated with declines in objective cognition (OC). A related but distinct construct is subjective cognition (SC), which is an individual’s self-appraisal of their OC. Research shows that SC impairment is an important precursor to declines in OC (Sánchez-Benavidez et al., 2018). Research has also demonstrated a positive relationship between OC and social support (SS) across adulthood (La Fleur & Salthouse, 2017), but there is limited research on the relationship between SC and SS. Participants (N = 1,873; age range 18-99) from the Virginia Cognitive Aging Project completed assessments of multiple domains of SC, OC, and SS. Results from the current study showed a consistent, significant association between negative interactions with others and poorer SC (Betas ranged from -.077 to .103, p < .05), beyond the influence of sociodemographic, well-being, and health factors. Our findings suggest that negative interactions may adversely impact one’s self-appraisal of cognitive functioning.

